# Supplementary material for: Association between the intake of dietary n3 and n6 fatty acids and stroke in US adults: A cross-sectional study of NHANES 2007–2018
Source: PLoS One. 2023 Nov 16;18(11):e0293893. doi: 10.1371/journal.pone.0293893 (PMC10653462; doi:10.1371/journal.pone.0293893)
Supplement: S1 Table — (DOCX) [file pone.0293893.s001.docx]

**Table S1 Detailed process of subjects exclusion**

| Step | The number of subjects before exclusion | The reasons of exclusion | NO.(subjects) | The number of subjects after exclusion |
| --- | --- | --- | --- | --- |
| 1 | 59842 | Age ＜18 | 23262 | 36580 |
| 2 | 36580 | missing stroke data (who lack information on the diagnosis of stroke) | 3256 | 33324 |
| 3 | 33324 | missing dietary data (participants with incomplete or unreliable 24-h recall dietary data) | 2886 | 30438 |
| 4 | 30438 | pregnant or lactating women | 469 | 29969 |
| 5 | 29969 | inappropriate energy intake (＜ 500 or [≥](https://baike.baidu.com/item/%E2%89%A5?fromModule=lemma_inlink) 5000 kcal/day for females, and ＜ 500 or [≥](https://baike.baidu.com/item/%E2%89%A5?fromModule=lemma_inlink) 8000 kcal/day for males) | 510 | 29459 |
