# Supplementary material for: Association between the intake of dietary n3 and n6 fatty acids and stroke in US adults: A cross-sectional study of NHANES 2007–2018
Source: PLoS One. 2023 Nov 16;18(11):e0293893. doi: 10.1371/journal.pone.0293893 (PMC10653462; doi:10.1371/journal.pone.0293893)
Supplement: S2 Table — (DOCX) [file pone.0293893.s002.docx]

**Table S1. The classifications of covariates**

| Covariates | Classification |
| --- | --- |
| Age | 18-39; 40-59; ≥60 |
| Sex | Male; Female |
| Race | Mexican American; Other Hispanic; Non-Hispanic White; Non-Hispanic Black; Other Race |
| Marital status | Married/living with partner; Widowed/divorced/separated; Never married |
| Education level | Below high school; High school; Above high school |
| Poverty-income ratio (PTR) | ≤0.99; and ≥1.00 |
| Body mass index (BMI) | ≤25kg/m^2^; 25-30kg/m^2^; ≥30kg/m^2^ |
| Work activity | Vigorous; Moderate; Other |
| Recreational activity | Vigorous; Moderate; Other |
| Drinking status^a^ | Yes; No |
| Smoking status^b^ | Yes; No |
| Sleeping disorder | Yes; No |
| Hypertension^c^ | Yes; No |
| Diabetes^d^ | Yes; No |
| Hypercholesterolemia^e^ | Yes; No |
| Energy intake^f^ | Continuous |

^a^ Drinking status was stratified according to whether they have drunk at least 12 times last year. ^b^ Smoking status was stratified according to whether they smoked at least 100 cigarettes in life.^c^ Systolic blood pressure (SBP) ≥130 mm Hg, or diastolic blood pressure (DBP) ≥80 mm Hg, or currently taking antihypertensive drugs were hypertensive patients. ^d^Diabetes was defined as self-reported physician diagnosis or taking insulin or anti-diabetic pills. ^e^ Hypercholesterolemia was defined as self-reported physician diagnosis or taking cholesterol-lowering drugs.^f^ Energy intake of each participant was obtained by summing the mean of the two 24h dietary intakes and the mean of two 24h intakes from supplements.
