# Supplementary material for: Association between the intake of dietary n3 and n6 fatty acids and stroke in US adults: A cross-sectional study of NHANES 2007–2018
Source: PLoS One. 2023 Nov 16;18(11):e0293893. doi: 10.1371/journal.pone.0293893 (PMC10653462; doi:10.1371/journal.pone.0293893)
Supplement: Attachment — Submitted filename: Response to Reviewers.docx [file pone.0293893.s004.docx]

**Response to Reviewers**

**Manuscript title:** Association of dietary n3, n6 fatty acids intake with stroke in US adults: a cross-sectional study of NHANES 2007-2018

**Manuscript ID:** PONE-D-23-13938

Comments to the Author

1. Is the manuscript technically sound, and do the data support the conclusions?

The manuscript must describe a technically sound piece of scientific research with data that supports the conclusions. Experiments must have been conducted rigorously, with appropriate controls, replication, and sample sizes. The conclusions must be drawn appropriately based on the data presented.

Reviewer #1: Yes

Reviewer #2: Partly

In our manuscript, we described a technically sound piece of scientific research and the data could support the conclusions.

2.Has the statistical analysis been performed appropriately and rigorously?

Reviewer #1: Yes

Reviewer #2: No

We have strictly followed the data processing methods of the NHANES database and applied the appropriate analysis methods and models, the same methods have been used in previous published articles.

3.Have the authors made all data underlying the findings in their manuscript fully available?

The PLOS Data policy requires authors to make all data underlying the findings described in their manuscript fully available without restriction, with rare exception (please refer to the Data Availability Statement in the manuscript PDF file). The data should be provided as part of the manuscript or its supporting information, or deposited to a public repository. For example, in addition to summary statistics, the data points behind means, medians and variance measures should be available. If there are restrictions on publicly sharing data—e.g. participant privacy or use of data from a third party—those must be specified.

Reviewer #1: Yes

Reviewer #2: Yes

We have made all data underlying the findings in our manuscript fully available.

4.Is the manuscript presented in an intelligible fashion and written in standard English?

PLOS ONE does not copyedit accepted manuscripts, so the language in submitted articles must be clear, correct, and unambiguous. Any typographical or grammatical errors should be corrected at revision, so please note any specific errors here.

Reviewer #1: No

Reviewer #2: Yes

In the revised manuscript, we have carefully corrected the errors.

5.Review Comments to the Author

Please use the space provided to explain your answers to the questions above. You may also include additional comments for the author, including concerns about dual publication, research ethics, or publication ethics. (Please upload your review as an attachment if it exceeds 20,000 characters)

Reviewer #1: The authors used the data from the 2007–2018 National Health and Nutrition Examination Survey (NHANES) to study the association between dietary n3, n6 fatty acids intake and stroke in a sample of 29,459 adults. They found that found that dietary n3 and n6 fatty acids intake were inversely associated with stroke in US adults. The novelty of the study is limited.

1. Abstract: The statement “The association between dietary n3, n6 fatty acids intake and the risk of stroke has not been investigated in the general population.” seems problematic. Supported by literature?

Reply: Thank you very much for your questions. It is imprudent for us to say so, and we have corrected it in the revised manuscript. Please seen line 24-26 on page 2 for detail.

2. Introduction: The sentence “Given the negative impact of stroke on individuals and society, it is important to find effective non-drug interventions to reduce the incidence of stroke [5].” appears meaningless, which should be deleted.

Reply: Thank you very much for your comments. We have deleted this sentence in the revised manuscript. Please seen line 58 on page 2 for detail.

3. The intake of fatty acids was not fully defined. Daily intake? The investigators seemed able to estimate the intake on a cross-sectional basis only, while the impact of fatty acids must be a long-term one. The authors are unable to explain why individuals at some certain age failed to present the inverse associations.

Reply: Thank you very much for your in-depth questions. It is daily dietary intake of n3, n6 fatty acids calculated in our study, and we have made it clear in the revised manuscript. Please seen line 109-110 on page 5 for detail. The NHANES database uses a method of 24-hour dietary review to assess the nutritional health status of residents, and long-term nutrient monitoring data were not available in this database. This is a major shortcoming of cross-sectional studies, and we have summarized it in the limitation section of the discussion. Please seen line 239-242 on page 24 for detail. In our study, we fail to find a significant negative association between dietary n3, n6 fatty acids intake and the risk of stroke in people aged 19-39 and 40-59, possibly because there are fewer stroke cases in the two age groups, thus weakening the association between dietary n3, n6 fatty acids and stroke. Moreover, we have explored the interaction of age in the relationship between dietary n3, n6 fatty acids and stroke in the revised manuscript. We found that there was an interaction between dietary n3 (P-interaction ＜0.001), n6 (P-interaction ＜0.001) fatty acids and age on stroke event. Please seen line 171-173 on page 16 and line 180-181 on page 19 for detail.

4. The authors are supposed to do stratified analyses, e.g. ischemic stroke and cerebral hemorrhage, because stroke per se is highly heterogeneous.

Reply: Thank you very much for your advice. Unfortunately, the NHANES database does not distinguish between types of stroke, so our study can only suggest an association between dietary n3, n6 fatty acids and stroke event, and does not clarify whether the stroke was haemorrhagic or ischemic.

5. Language：the manuscript is in need of English language editing. There appear many errors in English grammar, syntax, and word usage including, but are not limited to, lack of subject-verb agreement (singular vs. plural), inconsistent tense usage, improper word forms (noun vs. verb vs. adjective vs. gerund). Accordingly, the reviewer suggests that the authors have the text professionally edited by an English improvement service before the manuscript can be reconsidered for publication in the journal. For example, grammatical error is seen in the sentences “Stroke is also one of leading causes of long-term disability worldwide, leading a high health care expenditures and low quality of life for those survive from it[3].” “Herein, we use a large sample in the National Health and Nutrition Examination Survey (NHANES) from 2007-2018 to calculated the associations of dietary n3 and n6 fatty acids intake and the risk of stroke.”

Reply: Thank you very much for your advice. We have corrected the errors reduced the repetition rate in the revised manuscript. Please review it again.

Reviewer #2: In this study, the association between dietary n3 and n6 fatty acid intake and the risk of stroke was investigated. The authors concluded that dietary intake of n3 and n6 fatty acids might be inversely associated with the risk of stroke in US adults. However, there are some concerns.

1. As mentioned by the authors in the limitations section, the primary weakness of this study is its cross-sectional design, which precludes establishing causation. While the authors discuss potential causal relationships between dietary n3 and n6 fatty acid intake and stroke risk, this discussion might not be appropriate. The discussion should be grounded in the observed results, specifically that participants with stroke had lower dietary n3 and n6 fatty acid intake, whereas those without stroke had higher intake of these fatty acids. Therefore, the authors are likely unable to draw causal inferences regarding cause and effect.

Reply: Thank you very much for your in-depth questions. We have revised this limitation in the discussion section. Please seen line 236-239 on page 24 for detail.

2. Another notable limitation is the definition of the outcome, stroke, based on the Medical Conditions Questionnaire. This raises questions about its validity. Given the heterogeneous nature of stroke, standardized clinical criteria should ideally be used for diagnosis. The potential for misdiagnosis or recall bias due to this definition should be acknowledged. It is advisable to provide references that support the acceptability of this questionnaire-based definition.

Reply: Thanks for the advice.It is a pity that the NHANEA database does not include the stroke data from standardized clinical criteria. The measures of self-reported stroke by questionnaire are reliable and have been employed in many previous articles. In the revised manuscript, we have provided references to support this point. Please seen line 97-98 on page 5 for detail. Compared with the clinical standard diagnosis results, the stroke diagnosis obtained from the questionnaire may indeed lead to misdiagnosis and recall bias. We have summarized it in the limitation section. Please seen line 242-244 on page 24 for detail.

3. Were interactions examined in subgroup analyses by the authors? Any statistical differences observed within subgroups should be discussed in the context of the results.

Reply: Thanks for your suggestion. We have explored the interactions in subgroup analyses, and the results were showed in Table 3 and Table 4. The results indicated that subgroup analyses by sex showed no statistically significant interactions, while subgroup analyses by age showed a significant interactions. We have summarized these in the results. Please seen line 171-173 on page 16 and line 180-181 on page 19 for detail.

4. Could the exclusion of certain participants introduce selection bias? This possibility should be considered and addressed in the limitations section.

Reply: Thanks for your suggestion. We have added this in the limitation section.Please seen line 243-244 on page 24 for detail.

5. The study covers a 12-year span of data accumulation. Has there been any notable change in dietary n3 and n6 fatty acid intake or in the incidence of stroke during this period? Exploring such potential changes could provide valuable context to the study's findings.

Reply: Thanks for very much for your suggestion. We have analyzed the data from the six cycles, and the results are as follows (Table 1 and Figure1-2). From the results, we can found that dietary n3, n6 fatty acids intake shows an overall upward trend from 2007 to 2018, but the prevalence of stroke fluctuated. Therefore, we do not discuss this result in the manuscript.

**Table 1.** **Changes in dietary n3, n6 fatty acids intake and stroke prevalence from 2007 to 2018**

| Year | Number of participants | Dietary n3 fatty acids intake (m±SD） | Dietary n6 fatty acids intake (m±SD） | Stroke cases | Prevalence of stroke |
| --- | --- | --- | --- | --- | --- |
| 2007-2008 | 5151 | 1.49±0.99 g/day | 13.57±8.10 g/day | 217 | 4.21% |
| 2009-2010 | 5539 | 1.53±0.93 g/day | 13.97±8.21 g/day | 193 | 3.48% |
| 2011-2012 | 4629 | 1.77±1.08 g/day | 15.65±9.00 g/day | 174 | 3.76% |
| 2013-2014 | 4856 | 1.76±1.10 g/day | 15.43±8.77 g/day | 157 | 3.23% |
| 2015-2016 | 4776 | 1.73±1.15 g/day | 15.03±8.77 g/day | 169 | 3.54% |
| 2017-2018 | 4508 | 1.85±1.20 g/day | 16.45±9.55 g/day | 211 | 4.68% |

Figure 1. Changes in the prevalence of stroke from 2007 to 2018

|  | Prevalence of stroke |
| --- | --- |
| 2007-2008 | 4.21% |
| 2009-2010 | 3.48% |
| 2011-2012 | 3.76% |
| 2013-2014 | 3.23% |
| 2015-2016 | 3.54% |
| 2017-2018 | 4.68% |

Figure 2. Changes in dietary n3, n6 fatty acids intake from 2007 to 2018

|  | Dietary n3 fatty acids intake | Dietary n6 fatty acids intake |
| --- | --- | --- |
| 2007-2008 | 1.49 | 13.57 |
| 2009-2010 | 1.53 | 13.97 |
| 2011-2012 | 1.77 | 15.65 |
| 2013-2014 | 1.76 | 15.43 |
| 2015-2016 | 1.73 | 15.03 |
| 2017-2018 | 1.85 | 16.45 |
