# Supplementary material for: Association between the intake of dietary n3 and n6 fatty acids and stroke in US adults: A cross-sectional study of NHANES 2007–2018
Source: PLoS One. 2023 Nov 16;18(11):e0293893. doi: 10.1371/journal.pone.0293893 (PMC10653462; doi:10.1371/journal.pone.0293893)
Supplement: Attachment — Submitted filename: Response to Reviewers.docx [file pone.0293893.s005.docx]

**Response to Reviewers**

**Manuscript title:** Association of dietary n3, n6 fatty acids intake with stroke in US adults: a cross-sectional study of NHANES 2007-2018

**Manuscript ID:** PONE-D-23-13938

Journal Requirements:
Please review your reference list to ensure that it is complete and correct. If you have cited papers that have been retracted, please include the rationale for doing so in the manuscript text, or remove these references and replace them with relevant current references. Any changes to the reference list should be mentioned in the rebuttal letter that accompanies your revised manuscript. If you need to cite a retracted article, indicate the article’s retracted status in the References list and also include a citation and full reference for the retraction notice.

Reply: We have reviewed the list of reference and we did not cite retracted paper in my manuscript. But in this progress, we found same mistakes and we have corrected them in the revised manuscript. Please seen Line 297, Line 300-301, Line 338 in Page 26-27.

The manuscript requires thorough language editing, preferably by a native speaker, to ensure the best quality. Additionally, the Discussion section needs to be revised to accurately reflect the association between fatty acid intake and stroke risk, but not a causal relationship. Furthermore, a paragraph should be added to describe the differences and mechanisms observed in stroke risk across different age groups. It is important to include a limitation section acknowledging the heterogeneity of stroke outcomes and the inability to stratify between ischemic and hemorrhagic strokes.

Reply: The language of the manuscript was edited throughout, and we also revised the section of discussion, please seen Line 213-219 in Page 22. In the revised manuscript, a paragraph has been added to describe the differences and mechanisms observed in stroke risk across different age groups. Please seen Line 250-260 in Page 24. We have added the disadvantage of not being able to stratifying ischemic and hemorrhagic strokes, Please seen Line 275-277 in Page 25.
